# ω-6 Polyunsaturated Fatty Acids and Cardiometabolic Health: Current Evidence, Controversies, and Research Gaps

**DOI:** 10.1093/advances/nmy038

**Published:** 2018-09-04

**Authors:** Kevin C Maki, Fulya Eren, Martha E Cassens, Mary R Dicklin, Michael H Davidson

**Affiliations:** 1 Midwest Biomedical Research: Center for Metabolic and Cardiovascular Health, Glen Ellyn, IL; 2 ACH Food Companies, Inc., Oakbrook Terrace, IL; 3 Department of Medicine, Section of Cardiology, University of Chicago, Chicago, IL

**Keywords:** PUFAs, omega-6 fatty acids, linoleic acid, SFAs, cardiometabolic risk, cardiovascular disease, diabetes, dietary guidelines

## Abstract

The 2015 Dietary Guidelines for Americans recommend limiting the intake of saturated fatty acids (SFAs) to <10% of energy/d and replacing dietary SFAs with unsaturated fatty acids. A Presidential Advisory from the American Heart Association recently released its evaluation of the relation between dietary fats and cardiovascular disease (CVD), and also recommended a shift from SFAs to unsaturated fatty acids, especially polyunsaturated fatty acids (PUFAs), in conjunction with a healthy dietary pattern. However, the suggestion to increase the intake of PUFAs in general, and omega-6 (n–6) PUFAs in particular, continues to be controversial. This review was undertaken to provide an overview of the evidence and controversies regarding the effects of ω-6 PUFAs on cardiometabolic health, with emphasis on risks and risk factors for CVD (coronary heart disease and stroke) and type 2 diabetes mellitus (T2D). Results from observational studies show that higher intake of ω-6 PUFAs, when compared with SFAs or carbohydrate, is associated with lower risks for CVD events (10–30%), CVD and total mortality (10–40%), and T2D (20–50%). Findings from intervention studies on cardiometabolic risk factors suggest that ω-6 PUFAs reduce concentrations of LDL cholesterol and non-HDL cholesterol in a dose-dependent manner compared with dietary carbohydrate, and have a neutral effect on blood pressure. Despite the concern that ω-6 fatty acids increase inflammation, current evidence from studies in humans does not support this view. In conclusion, these findings support current recommendations to emphasize consumption of ω-6 PUFAs as a replacement of SFAs; additional randomized controlled trials with cardiometabolic disease outcomes will help to more clearly define the benefits and risks of this policy.

## Introduction

According to the AHA's 2018 Heart Disease and Stroke Statistics Update, cardiovascular disease [CVD; including coronary artery disease (CAD), hypertension, and stroke] is the number 1 cause of death in the United States, accounting for ∼1 of every 3 deaths (1). The 2015 Dietary Guidelines Advisory Committee reported that CVD was estimated to affect ∼35% of the US population in 2010, and 50% of normal weight and 75% of overweight/obese Americans were said to have at least 1 risk factor placing them at higher cardiometabolic risk, such as high blood pressure, smoking, or dyslipidemia (2). Furthermore, it is estimated that 9.1% of American adults have been diagnosed with diabetes, 3.1% have undiagnosed diabetes, and 33.9% have prediabetes (1). Thus, cardiometabolic diseases [particularly CVD and type 2 diabetes (T2D)] are leading causes of morbidity and mortality in the United States and worldwide. There is strong evidence that risks of these conditions are affected by lifestyle factors such as unhealthy eating patterns, inadequate physical activity, and cigarette smoking (3–9).

The Dietary Guidelines for Americans (DGA) 2015–2020 report states that the average American diet contains higher than optimal intakes of SFAs, added sugars, and refined starches (2). Nutrients that could be consumed as partial replacements for SFAs and refined carbohydrates (added sugars and refined starches) include protein, unsaturated fatty acids (UFAs), carbohydrates from unrefined sources, such as whole grains and legumes, and alcohol (consumed in moderation). The 2015 DGA recommend limiting the intake of SFAs to <10% of energy/d by replacing them with UFAs, while maintaining a total dietary fat intake of 20–35% (for men and women ≥19 y of age) (2, 10). This recommendation was based on what has been described as a strong and consistent body of evidence showing that replacement of SFAs with UFAs, especially PUFAs, is associated with decreased circulating concentrations of total cholesterol (TC) and LDL cholesterol, and with reduced risk of CVD events and CVD-related deaths in observational studies (2).

The strongest evidence for assessing the effects of a dietary exposure or modification and disease risk is the randomized controlled trial (RCT). Unfortunately, few RCTs have been completed to evaluate the effects of increasing intakes of UFAs as a replacement for other dietary components such as SFAs or refined carbohydrates on cardiometabolic disease outcomes. Of the RCTs that are available, most were completed in the 1960s and 1970s, were relatively small, and had a number of other limitations such as high rates of attrition.

There is observational evidence reporting a positive association between SFA intake and atherosclerosis dating back to as early as the mid-1950s when Ancel Keys and colleagues first reported the results from their examination of diet and health in 7 countries that showed a positive correlation between high SFA intake and CAD, as well as a relation between blood cholesterol concentrations and the types of fatty acids ingested (11, 12). A Presidential Advisory from the AHA recently released its evaluation of the relation between dietary fats and CVD (13). They concluded that prospective observational studies have consistently found a lower risk of CAD, as well as CVD and all-cause mortality, with lower SFA consumption and higher intakes of PUFAs or MUFAs.

PUFAs include mainly omega-3 and omega-6 fatty acids. ω-3 PUFAs, particularly the marine-based fatty acids of EPA (20:5 ω-3 fatty acid) and DHA (22:6 ω-3 fatty acid), have been shown to possess a variety of potentially cardioprotective effects, including effects on blood lipids, hemodynamics, platelets, and fibrinolysis, and markers of inflammation and oxidative stress (14–18). This makes the increased consumption of ω-3 fatty acids as a replacement of SFAs an attractive approach, particularly because the average American diet has less than the recommended intake of 8 oz seafood/wk. However, ω-3 fatty acids are a quantitatively small portion of the diet, and it would be difficult to increase their consumption to the level necessary to substantially replace calories from other nutrients, particularly SFAs, but also refined starches and added sugars. ω-3 Fatty acid supplements are available and prescription ω-3 fatty acid products are approved by the FDA for the management of hypertriglyceridemia (14–17).

In contrast to ω-3 fatty acids, ω-6 fatty acids are a large component of the commonly used oils in the American diet, and represent a more feasible replacement for SFAs. The predominant ω-6 fatty acid is the essential fatty acid linoleic acid (LA; 18:2 ω-6 fatty acid); α-linolenic acid (ALA; 18:3 ω-3 fatty acid) is the other essential fatty acid. LA accounts for 80–90% of total dietary PUFAs (19). Typical dietary intakes of LA in the United States are ∼6% of energy (20). Soybean oil (usually labeled as vegetable oil), sunflower oil, and corn oil are all high in ω-6 PUFAs (**Table 1**) (13). Qualified health claims exist for soybean, canola, corn, and olive oils for their potential abilities to reduce the risk of heart disease (21–24).

**TABLE 1:** Estimated fatty acid content of commonly consumed cooking oils or solid fats^1^

	SFAs, g/100 g			MUFAs, g/100 g		PUFAs, g/100 g		
Fats/oils	Total	12:0 14:0 16:0	18:0	Total	18:1	Total	18:2n–6	18:3n–3
Dairy fat (butter)	63	39	12	26	21	4	3	0
Tallow (beef)	50	30	19	42	36	4	3	1
Lard (pork)	39	25	14	45	41	11	10	1
Coconut oil	82	67	3	6	6	2	2	0
Palm kernel oil	82	72	3	11	11	2	2	0
Palm oil	49	45	4	37	37	9	9	0
Peanut oil	17	10	2	46	45	32	32	0
Olive oil^2^	14	11	2	73	71	10	10	1
Canola oil^2^	7	4	2	63	62	28	19	9
Soybean oil^2^	16	10	4	23	23	58	50	7
Corn oil^2^	13	11	2	28	27	55	53	1
Sunflower oil (high linoleic)	10	6	4	20	20	66	66	0
Sunflower oil (high oleic)	10	5	4	84	83	4	4	0
Safflower oil (high linoleic)	6	4	2	14	14	75	75	0
Safflower oil (high oleic)	8	5	2	75	75	13	13	1

^1^A 0 value equals <0.5 g/100 g. Adapted from Sacks et al. (13) with permission; data from USDA food composition tables.

^2^Qualified health claims (21–24).

Despite the fact that most dietary recommendations agree that SFAs should be at least partially replaced by UFAs, in particular vegetable PUFAs, the suggestion to increase the intake of PUFAs in general, and ω-6 PUFAs in particular, continues to be controversial (2, 13, 25).

## Methods

This editorial review was undertaken to present a balanced overview of the controversies and evidence regarding the potential benefits and risks of consumption of ω-6 PUFAs on cardiometabolic health, with a focus on incident CVD and T2D, as well as biomarkers of cardiometabolic disease risk, including lipoprotein lipids, blood pressure, insulin sensitivity, and inflammation. The review focused on the evidence considered by the DGA 2015 (2) and the 2017 AHA Presidential Advisory on Dietary Fats and Cardiovascular Disease (13), new results published since that time, and an appraisal of dissenting viewpoints expressed by experts in the field of diet and cardiometabolic health. Instances throughout the paper when specifically ω-3 or ω-6 PUFAs are being described are labeled as such. Statements that refer generally to PUFAs or UFAs do not specifically call out ω-3 or ω-6 fatty acids.

## RCT evidence for **ω**-6 PUFAs and risks for incident CVD, CAD, and CVD mortality

Data from RCTs are considered by many to represent the highest-quality evidence for use in developing dietary recommendations (25, 26). However, RCTs examining the effects of dietary fatty acids on disease are often difficult to perform and interpret because of the relatively crude methods used to assess background diet, difficulties with long-term adherence necessary to test dietary interventions, and variations among subjects with regard to disease severity and the presence of phenotypic and genotypic variants that can affect the metabolism of fatty acids (27).

**FIGURE 1:**
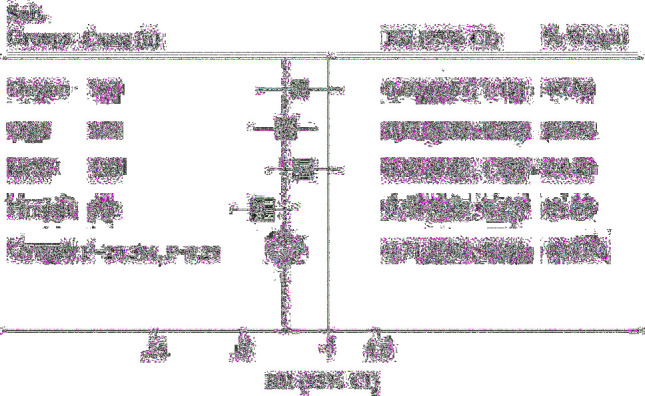
Meta-analysis of core clinical trials replacing SFAs with PUFAs assessed for the Presidential Advisory from the AHA statement on dietary fats and cardiovascular disease. RR values and 95% CIs are for the primary coronary heart disease outcome for each trial. MRC, Medical Research Council. Reproduced from Sacks et al. (13) with permission.

Unfortunately, most of the RCT data available from direct examination of interventions intended to alter dietary fatty acid intakes to evaluate their effects on cardiometabolic disease are quite old. The AHA Presidential Advisory recently reported its meta-analysis findings that showed a 29% reduction in CAD events from replacing SFAs with PUFAs (RR: 0.71; 95% CI: 0.62, 0.81) (**Figure 1**) (13). That analysis emphasized results from 4 core RCTs, all completed decades ago, that met their criteria of at least 2 y duration and good adherence as indicated by blood or tissue concentrations of cholesterol and/or PUFAs (13, 28–33). The AHA Advisory statement has been criticized by some because of its limitation to 4 RCTs. A comprehensive meta-analysis of 15 RCTs performed by Mozaffarian et al. (34) also reported a significant reduction in total CAD risk with replacement of 5% of SFAs by PUFAs, but the magnitude of the reported effect was much smaller (RR: 0.90; 95% CI: 0.83, 0.97) than that reported by the AHA Presidential Advisory. In contrast, a meta-analysis of observational studies and RCTs by Chowdhury et al. (35) reported that in 8 RCTs ω-6 PUFA intake produced a larger reduction in coronary risk than that seen with ALA or ω-3 PUFA supplementation, but the decrease did not reach statistical significance (RR: 0.89; 95% CI: 0.71, 1.12).

A Cochrane meta-analysis by Hooper et al. (36) examined the effect of reducing SFA intake by replacing it with carbohydrate, PUFA, or MUFA and/or protein and reported that such replacements reduced the risk of cardiovascular events by 17% (95% CI: 0.72, 0.96). Subgroup analyses suggested that the reduction was due to those studies that primarily replaced SFAs with PUFAs. They reported that “replacing some saturated fat with PUFA”, predominantly of plant origin, had the following effects: all-cause mortality (7 studies; RR: 0.96; 95% CI: 0.82, 1.13), cardiovascular mortality (7 studies; RR: 0.95; 95% CI: 0.73, 1.25), cardiovascular events (7 studies; RR: 0.73; 95% CI: 0.58, 0.92), fatal and nonfatal myocardial infarction (7 studies; RR: 0.83; 95% CI: 0.67, 1.02), nonfatal myocardial infarction (5 studies; RR: 0.80; 95% CI: 0.63, 1.03), stroke (4 studies; RR: 0.68; 95% CI: 0.37, 1.27), CAD mortality (7 studies; RR: 0.98; 95% CI: 0.74, 1.28), and CAD events (7 studies; RR: 0.76; 95% CI: 0.57, 1.00). Thus, although all of the subgroup analyses showed pooled RR estimates below 1.0 (i.e., lower risk in PUFA compared with SFA groups), only 1 of 8 had a 95% CI with an upper limit below 1.0, indicating statistical significance at the traditional level of 0.05.

**FIGURE 2:**
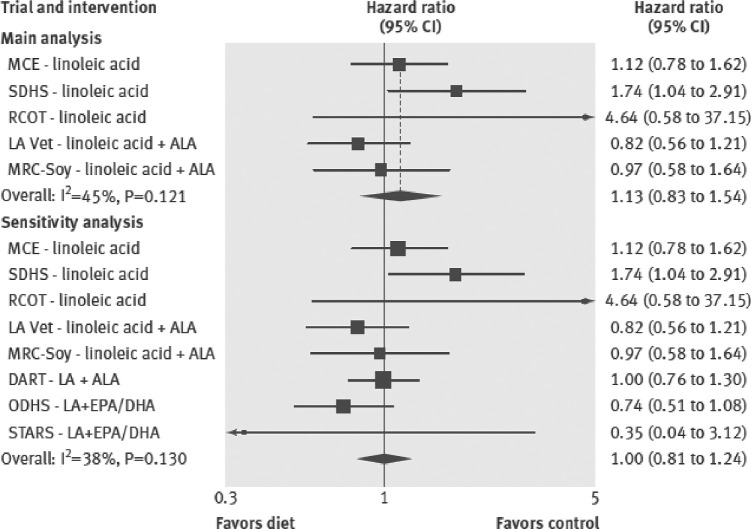
Meta-analysis for mortality from coronary heart disease from trials replacing SFA with vegetable oils rich in linoleic acid. ALA, alpha-linolenic acid; DART, Diet and Re-infarction Trial; LA, linoleic acid; LA Vet, Los Angeles Veterans Trial; MCE, Minnesota Coronary Experiment; MRC-Soy, Medical Research Council Soy Oil Trial; ODHS, Oslo Diet Heart Study; RCOT, Rose Corn Oil Trial; SDHS, Sydney Diet Heart Study; STARS, St. Thomas Atherosclerosis Regression Study. Reproduced from Ramsden et al. (38) with permission.

Because few RCTs have been published that tested the cause-effect relation between reducing SFAs and effects on heart disease, Ramsden et al. recently investigated previously unpublished data from the Sydney Diet Heart Study (1966–1973) (37) and the Minnesota Coronary Survey (1968–1973) (38) that both replaced SFAs with vegetable oil rich in LA. Results from a meta-analysis that included data recovered from these trials, as well as 3 additional RCTs, indicated that there was no evidence of benefit on CAD mortality (HR: 1.13; 95% CI: 0.83, 1.54) or all-cause mortality (HR: 1.07; 95% CI: 0.90, 1.27) (**Figure 2**) (38). However, inclusion of results from the Minnesota Coronary Survey of patients hospitalized for mental illness is controversial for several reasons related to its study design, including relatively short duration of subject enrollment, high rate of withdrawals, and intermittent treatment administration (13). Results from the Sydney Diet Heart Study are also controversial because subjects assigned to the high-PUFA diet received a margarine that was high in *trans* UFAs, which has since been acknowledged to be harmful and is being removed from the food supply (13).

A recent meta-analysis by Hamley (39) that examined the effect of replacing SFAs with PUFAs on CAD risk pointed to the importance of considering whether the trials included in a meta-analysis have been adequately controlled. Clinical trials that the authors categorized as adequately controlled were those that most accurately tested the effect of replacing SFAs with mostly ω-6 PUFAs, whereas clinical trials categorized as inadequately controlled had additional dietary and/or nondietary differences between groups that did not allow direct comparison or clearly unconfounded comparison of SFAs with ω-6 PUFAs. Examples of these differences include inclusion or noninclusion of *trans* fatty acids, multifactorial dietary interventions, varying vitamin E intakes, and use of cardiotoxic medications. Among the 5 trials classified as adequately controlled, there was no effect of mostly ω-6 PUFAs on major CAD events, total CAD events, CAD mortality, and total mortality (RRs range: 1.02–1.13). However, when the pooled results from all trials, including the 6 trials classified as inadequately controlled, were examined, there was a suggestion that replacing SFAs with mostly ω-6 PUFAs significantly reduced the risk of total CAD events (RR: 0.80; 95% CI: 0.65, 0.98; *P* = 0.03), but not major CAD events (RR: 0.87; 95% CI: 0.70, 1.07), CAD mortality (RR: 0.90; 95% CI: 0.70, 1.17), or total mortality (RR: 1.00; 95% CI: 0.90, 1.10).

Thus, in our view, the data from RCTs regarding the replacement of SFAs with ω-6 PUFAs on CVD outcomes suggest potential benefits, but are far from conclusive. It is especially difficult to interpret the findings since the trials were generally completed many years ago when the age-adjusted risks of CVD events in the populations in the United States and Europe were higher than they are today, background diets were quite different from current diets (higher in SFA intake, lower in refined sugars, refined grains, and refined vegetable oils), the prevalence of obesity was considerably lower, that of cigarette smoking was higher, and there was less routine use of preventive therapies such as blood pressure- and cholesterol-lowering drug therapies. Well-designed and properly controlled RCTs are needed to more clearly elucidate the potential cardiometabolic benefits and possible risks of replacing SFAs (and refined carbohydrates) with ω-6 PUFAs.

## Epidemiologic evidence for effects of higher **ω**-6 PUFA intake on cardiometabolic health

As mentioned previously, compared with RCTs, there is a large body of data from prospective observational studies investigating the associations between dietary fatty acids and cardiometabolic disease incidence. These have an important place in examining diet and disease relations, but there are limitations of this approach when it comes to informing decisions regarding dietary recommendations (25, 26). Interpretation of the results from prospective observational studies is difficult for a variety of reasons. Dietary assessment methods are subject to a variety of random and nonrandom sources of error. In addition, intakes of nutrients or dietary components are often highly correlated with other dietary components, as well as a variety of other characteristics that have the potential to produce interactions and/or residual confounding, even with extensive multivariate adjustment. Also, foods high in energy from a macronutrient such as ω-6 PUFAs will, by definition, displace other foods in the diet with a different nutritional profile. Thus, the apparent effects of a dietary exposure may, at times, reflect the influence of reducing the dietary components replaced (26).

Another consideration is that it is particularly difficult to separate the effects or associations with LA (ω-6 fatty acid) from those with ALA (ω-3 fatty acid), because they are largely consumed in the same plant oils and foods (collinearity), albeit LA is usually in higher amounts. Observational studies do not provide definitive evidence of a cause and effect relation between dietary intake and disease because of the potential for confounding and bias (26, 40). However, observational data are critically important because they examine people in more natural circumstances as opposed to the closely controlled environment of RCTs and contribute importantly to the body of evidence assessing the effects of dietary exposures on disease risk.

In a meta-analysis of 28 prospective cohort studies, Skeaff and Miller (41) reported that a higher compared with lower PUFA intake was not significantly associated with CAD events (RR: 0.97; 95% CI: 0.74, 1.27; *P* = 0.825), and similarly higher LA intake was not significantly associated with CAD events (RR: 1.05; 95% CI: 0.92, 1.20; *P* = 0.474). However, higher compared with lower PUFA intake was significantly associated with CAD mortality (RR: 1.25; 95% CI: 1.06, 1.47; *P* = 0.009). In their meta-analysis of 32 observational studies, Chowdhury et al. (35) also reported that there was no significant relation when comparing risk for coronary disease between the top and bottom tertiles of baseline ω-6 PUFA intake (RR: 0.98; 95% CI: 0.90, 1.06).

In contrast, meta-analyses that have specified the replacement nutrient in their modeling have indicated a significantly lower CAD risk when PUFAs partially replaced calories from SFAs, but not when the replacement nutrient was carbohydrate (i.e., low-fat diets) (42, 43). In a meta-analysis of data from 11 American and European cohort studies, Jakobsen et al. (42) reported a 13% reduction in risk of coronary events and a 26% reduction in risk of coronary death (HR: 0.74; 95% CI: 0.61, 0.89) when PUFAs replaced 5% of energy intake from SFAs (HR: 0.87; 95% CI: 0.77, 0.97). A meta-analysis by Farvid et al. (43) of 13 published and unpublished cohort studies found that a 5% energy increment in LA intake, to replace energy from SFAs, was associated with a 9% lower risk of CAD events (RR: 0.91; 95% CI: 0.87, 0.96) and a 13% lower risk of CAD deaths (RR: 0.87; 95% CI: 0.82, 0.94).

Results from recently published prospective cohort studies are generally supportive of the results from the published meta-analyses. An examination of participants in the Nurses’ Health Study (female nurses) and the Health Professionals Follow-up Study (male health professionals) showed, based on modeling, that replacing 5% of SFAs with equivalent energy intake from PUFAs was associated with a 25% lower risk of CAD (HR: 0.75; 95% CI: 0.67, 0.84; *P* < 0.0001) (44). Wang et al. (45) reported in this same cohort a 27% reduction in total mortality (HR: 0.73; 95% CI: 0.70, 0.77) when 5% of energy from SFAs was replaced with equivalent energy from PUFAs and a 13% reduction in total mortality (HR: 0.87; 95% CI: 0.82, 0.93) when SFAs were replaced with MUFAs. A positive association with cardiovascular mortality was found when 5% of carbohydrate was replaced with SFAs (HR: 1.08; 95% CI: 1.04, 1.11; *P* < 0.001), and inverse associations when 5% of carbohydrate was replaced with PUFAs (HR: 0.72; 95% CI: 0.65, 0.80; *P* < 0.001) or with MUFAs (HR: 0.96; 95% CI: 0.84, 1.09; *P* < 0.001) (44). Another recent prospective cohort investigation by Guasch-Ferré et al. (46) among participants in the Prevención con Dieta Mediterránea (PREDIMED) study found that modeling the replacement of 5% of energy from SFAs with PUFAs was associated with a 33% lower risk of incident CVD (HR: 0.67; 95% CI: 0.45, 0.98) and a 39% lower risk of all-cause death (HR: 0.61; 95% CI: 0.39, 0.97).

Most recently, a risk assessment model that included NHANES data from 1999–2002 and 2009–2012 was used to examine associations of consumption of 10 foods/nutrients, including PUFAs, with cardiometabolic mortality in 2012 (47). Optimal consumption, defined as the observed level at which the lowest risk occurred, of PUFAs as a percentage of energy replacing carbohydrates or SFAs was reported to be 11% for CAD risk reduction. Per 5% of PUFA energy/d replacing carbohydrates or SFAs, the RR (95% CI) for CAD among adults (≥25 y of age) was 0.88 (0.75, 0.94) for those 50 y of age and 0.92 (0.88, 0.96) for those 70 y of age. It was also estimated that 2.3% of the total US cardiometabolic deaths and 4.3% of the US CAD deaths in 2012 were associated with suboptimal consumption of PUFAs (<11% of PUFA energy/d replacing carbohydrates or SFAs).

We also conducted an investigation of the predicted effects on mortality of 1 tablespoon of liquid oils or butter replacing carbohydrate with the use of modeling equations based on the HRs reported by Wang et al. (45). The predicted effects of 1 tablespoon (13.5 g) cooking oils/d in a 2000 kcal/d diet, compared with carbohydrates, on mortality risk were: corn oil (−20.7%), canola oil (−16.2%), soybean (or vegetable) oil (−16.9%), and olive oil (−11.4%). The predicted effect of butter was to raise mortality risk by 2.4%. These effects are quantitatively rather large, and a portion of the effect could very well be due to confounding. Nevertheless, they are suggestive that additional research into the effects on mortality of replacing carbohydrates with PUFAs in the diet is warranted.

The Prospective Urban Rural Epidemiology (PURE) study is the largest observational study conducted to date that assessed the link between nutrient intakes, food group intakes, CVD events (including death), and overall mortality (48). It included data from >135,000 participants in 18 low-, middle-, and high-income countries across 5 continents. During the median follow-up of 7.4 y there were 5796 deaths and 4784 major CVD events. Results indicated that total fat, as well as SFA, MUFA, and PUFA intakes were significantly associated with lower risk for mortality. The HR for the highest compared with the lowest quintile of intake was 0.77 (95% CI: 0.67, 0.87) for total fat, 0.86 (95% CI: 0.76, 0.99) for SFAs, 0.81 (95% CI: 0.71, 0.92) for MUFAs, and 0.80 (95% CI: 0.71, 0.89) for PUFAs. For major CVD, the highest compared with the lowest quintile of SFA intake was associated with a significantly lower risk of stroke (HR: 0.79; 95% CI: 0.64, 0.98). Neither total fat nor any of the individual fats was associated with myocardial infarction risk or CVD mortality. These results challenge the current dietary recommendations’ emphasis on reducing SFA intake. However, SFA intake was generally low, with mean values ranging from 5.7% in China to 10.9% in Europe and North America. Across countries, total and SFA intakes are positively associated with socioeconomic status. Thus, in countries with higher intakes of total fat and SFAs, and thus lower carbohydrate intake, the higher socioeconomic status was likely associated with improved access to higher-quality health care, potentially confounding these results.

Because of the difficulties in determining dietary intakes of specific fatty acids, some investigations have utilized biomarkers of fatty acid concentrations such as in adipose tissue, plasma, plasma phospholipids, cholesterol esters, and erythrocyte membranes. These biomarkers are influenced by both dietary intake and endogenous metabolism of fatty acids (49). In the study by Chowdhury et al. (35) mentioned previously, there was no significant association detected between CAD and blood concentrations of ω-6 PUFAs (RR: 0.94; 95% CI: 0.84, 1.06 for the top compared with bottom tertiles), which was similar to the findings from dietary intake analyses. An evaluation of circulating ω-6 PUFAs in the Multi-Ethnic Study of Atherosclerosis (MESA) also failed to detect a significant association with CVD for ω-6 PUFAs [LA and arachidonic acid (AA)] (50). However, an examination of plasma phospholipid ω-6 PUFAs in older adults free of CVD in the Cardiovascular Health Study reported that higher LA was associated with a 13% lower risk of total mortality in the top compared with the bottom quintile (HR: 0.87; 95% CI: 0.74, 1.02; *P*-trend = 0.005), and that lower mortality was largely attributable to less mortality from CVD causes (51).

## Epidemiologic evidence for PUFAs (ω-6 fatty acids) and risk for T2D

**FIGURE 3:**
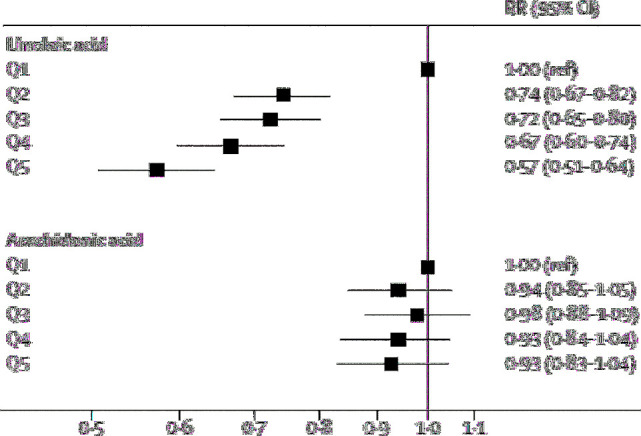
Pooled RRs of type 2 diabetes per quintile of linoleic acid and arachidonic acid biomarker from a meta-analysis of association between linoleic acid and type 2 diabetes assessed in multivariable models for each cohort. Q, quintile; ref, referent. Reproduced from Wu et al. (56) with permission.

Results from prospective cohort studies indicate a possible inverse relation between total ω-6 PUFA intake and risk of T2D status, but the magnitude and direction of the relation appear to vary among individual ω-6 fatty acids (52–56). A recent large pooled analysis of data from 20 prospective cohort studies with 39,740 adults from 10 countries (Iceland, Netherlands, the United States, Taiwan, the United Kingdom, Germany, Finland, Australia, Sweden, and France) examined the RR of T2D according to concentrations of LA and AA biomarkers (56). The investigators reported that in multivariate-adjusted, pooled analyses a higher proportion of LA biomarkers as percentage of total fatty acids was associated with lower risk of T2D overall (RR: 0.65; 95% CI: 0.60, 0.72, *P* < 0.0001) (**Figure 3**). This association was generally similar in phospholipid, plasma, cholesterol ester, and adipose tissue compartments. There was no significant association between AA biomarkers and T2D risk (RR: 0.96; 95% CI: 0.88, 1.05, *P* = 0.38). Furthermore, these relations were not significantly modified by age, BMI, sex, race, aspirin use, ω-3 PUFA concentrations, or variants of the *FADS* gene. These results confirmed those from another large prospective study on blood PUFAs and T2D risk in a pooled analysis of data from 8 European countries, the European Prospective Investigation into Cancer and Nutrition (EPIC)-InterAct Study (54). The investigators of EPIC-InterAct reported a strong inverse association between LA and risk of T2D (per 1 SD; HR: 0.80; 95% CI: 0.77, 0.83) and no relation with AA, but positive relations, with HRs ranging from 1.13 to 1.46 per 1 SD, between T2D risk and 4 other ω-6 fatty acids, including 18:3, 20:3, 22:4, and 22:5 (54).

The prospective Kuopio Ischaemic Heart Disease Risk Factor Study reported reduced T2D risk with higher concentrations of total serum ω-6 PUFAs (HR: 0.54; 95% CI: 0.41, 0.73; *P*-trend <0.001), LA (HR: 0.52; 95% CI: 0.39, 0.70; *P*-trend <0.001), and AA (HR: 0.62; 95% CI: 0.46, 0.85; *P*-trend = 0.007), but increased T2D risk with higher concentrations of gamma-linolenic acid (HR: 1.28; 95% CI: 0.98, 1.68, *P*-trend = 0.021) and dihomo-gamma-linolenic acid (HR: 1.38; 95% CI: 1.04, 1.84; *P*-trend = 0.005) (55). However, another recent prospective examination from the Finnish Diabetes Prevention Study failed to find a consistent association between LA and T2D risk among overweight patients with impaired glucose tolerance (57). RCTs examining the effects of ω-6 PUFAs on T2D risk, per se, are needed, although there have been several RCTs of ω-6 PUFA effects on biomarkers of T2D, such as insulin sensitivity and glycated hemoglobin (HbA1c), as described later in this paper.

## Effects of **ω**-6 PUFAs on biomarkers of cardiometabolic disease risk

### Lipoprotein lipids

Although there is a paucity of recent RCT data examining the effects of dietary fats on the risk for cardiometabolic disease outcomes, there is a large body of RCT data examining the effects of dietary fats on biomarkers of heart disease and diabetes. An elevated concentration of atherogenic cholesterol, i.e., the cholesterol carried in LDL and other apolipoprotein B–containing particles (non-HDL), is a major cause of atherosclerosis (58). Guidelines and recommendations universally include lowering an elevated concentration of LDL cholesterol as a target for CAD/CVD prevention, and some also include non-HDL cholesterol as a treatment target (58, 59). Many studies have investigated the effects on the blood lipoprotein profile of the main fatty acid classes of SFAs, MUFAs, and PUFAs (34, 60–66). Replacement of SFAs with PUFAs has been shown to lower LDL cholesterol and the TC:HDL cholesterol ratio (**Table 2**) (63).

**TABLE 2:** Estimated mean changes and 95% CIs in lipoprotein lipids for each 1% of dietary energy as SFAs isocalorically replaced with CHO, MUFAs, or PUFAs from an analysis of 74 trials^1^

	Change (mmol/L) per 1% energy replaced^2^		
Lipoprotein lipid	SFAs → CHO	SFAs → MUFAs	SFAs → PUFAs
Total-C	−0.041 (−0.047, −0.035)	−0.046 (−0.051, −0.040)	−0.064 (−0.070, −0.058)
LDL cholesterol	−0.033 (−0.039, −0.027)	−0.042 (−0.047, −0.037)	−0.055 (−0.061, −0.050)
HDL cholesterol	−0.010 (−0.012, −0.008)	−0.002 (−0.004, 0.000)	−0.005 (−0.006, −0.003)
Total-C/HDL cholesterol	0.001 (−0.006, 0.007)	−0.027 (−0.033, −0.022)	−0.034 (−0.040, −0.028)
TGs	0.011 (0.007, 0.014)	−0.004 (−0.007, −0.001)	−0.010 (−0.014, −0.007)

^1^Data are from a 2016 WHO systematic review and regression analysis report by Mensink (63). CHO, carbohydrate; Total-C, total cholesterol.

^2^All mean changes were statistically significant (*P* < 0.05) with the exception of the change in TG from replacement of SFA with CHO. To convert cholesterol from mmol/L to mg/dL multiply by 38.7; to convert TG from mmol/L to mg/dL multiply by 88.6.

LA and ALA are present together in many of the same vegetable oils and foods, but the proportion of LA is generally much higher than ALA (62, 63). LA and ALA, on a per-gram basis, appear to have similar effects on the lipoprotein lipid profile (67, 68). There are few studies of the effects of ω-6 fatty acids alone on blood lipids. A Cochrane review by Al-Khudairy et al. (69) compared the effects on blood lipids of increased ω-6 PUFAs with control in 3 trials (70–72). They reported no significant effects on TC (mean difference 0.02; 95% CI: −0.13 to 0.18), LDL cholesterol (mean difference −0.01, 95% CI: −0.14 to 0.12), HDL cholesterol (mean difference 0.01, 95% CI: −0.04 to 0.06), or TG (mean difference 0.03, 95% CI: −0.07 to 0.12). However, the results were dominated by 1 trial (72).

Soybean oil (vegetable oil), sunflower oil, and corn oil are all high in ω-6 PUFAs (Table 1). In clinical trials, consumption of corn oil, which contains ∼50–55% fatty acids as ω-6 PUFAs, has been shown to reduce LDL cholesterol concentrations (65, 67, 73). In a controlled study, Maki et al. (65) compared the effects of PUFA-rich corn oil with those of extra-virgin olive oil (higher in oleic acid) on plasma lipids and lipoproteins in men and women with elevated LDL cholesterol [≥130 mg/dL (3.4 mmol/L) and <200 mg/dL (5.2 mmol/L)]. After 21 d of consuming 4 tablespoons/d of foods made with either corn oil or olive oil, LDL cholesterol was decreased by 10.9% in the corn oil condition compared with 3.5% in the olive oil condition. A systematic review of 31 RCTs concluded that canola oil reduced LDL cholesterol, but had no effect on HDL cholesterol (74). RCTs of sunflower oil, rich in both MUFAs and ω-6 PUFAs and low in SFAs, have demonstrated that it also lowers LDL cholesterol, but the effect on HDL cholesterol is unclear (75). The FDA decisions to grant qualified health claims for soybean, canola, corn, and olive oils took into consideration the abilities of these oils to lower circulating concentrations of atherogenic cholesterol (21–24).

We investigated the predicted effects of the fatty acid differences of commonly consumed fats and oils on LDL cholesterol concentrations using the Yu and Kris-Etherton equation (76). The USDA Food Composition Databases were the primary source for fatty acid contents (77). The predicted effects of 2 tablespoons (27 g) cooking oils/d in a 2000 kcal/d diet, compared with carbohydrates, on LDL cholesterol were: corn oil [−8.9 mg/dL (−0.23 mmol/L)], canola oil [−8.3 mg/dL (−0.21 mmol/L)], soybean (or vegetable) oil [−6.5 mg/dL (−0.17 mmol/L)], and olive oil [−5.7 mg/dL (−0.15 mmol/L)]. The predicted effect of butter was to raise LDL cholesterol by 7.9 mg/dL (0.20 mmol/L).

This 6–9 mg/dL (0.16–0.23 mmol/L) reduction in LDL cholesterol with liquid oils replacing carbohydrate would be predicted to lower CVD risk by 3.9–5.6% over approximately 5 y, according to the relation between LDL cholesterol lowering and CVD risk from a meta-analysis of statin trials by the Cholesterol Treatment Trialists’ Collaboration (78), whereas butter replacing carbohydrate (0.2 mmol/L LDL cholesterol elevation) would be expected to increase risk by 5.1%. Thus, replacing 27 g/d of butter with liquid oil would be expected to lower CVD risk by ∼10% over 5 y. The effect could be larger, perhaps by a factor of ≥2, if the effects are estimated over a longer timeframe, as seen in studies of genetic variants, where the impact of a given difference in LDL cholesterol is roughly 2.0–2.5 times what has been observed in RCTs of drug therapies [e.g., statins, ezetimibe, anacetrapib, proprotein convertase subtilisin kexin type 9 (PCSK9) inhibitors] over ∼5 y (79). It is also important to note that lifestyle counseling, including nutrition recommendations, is a key element of CVD prevention for patients at all CVD risk levels and represents a lower risk and lower-cost option compared with drug therapies (7).

### Blood pressure

A cross-sectional analysis of elderly adults found that a 2-SD increase in dietary LA was associated with a modest 1.4-mm Hg decrease in systolic blood pressure and a 0.9-mm Hg decrease in diastolic blood pressure (80). However, 2 subsequent observational studies in middle-aged and older women or adolescents did not confirm those earlier results (81, 82).

RCTs that examined the effects on blood pressure of replacing calories from one fatty acid class with another fatty acid class, or with carbohydrates, have generally shown no effect or only small effects on blood pressure (83–85). In the OmniHeart controlled feeding trial, a diet low in SFAs with higher intake of UFAs (mainly MUFAs) decreased blood pressure by ∼1–3 mm Hg, compared with a diet similarly low in SFAs with 10% less energy from UFAs and 10% more from carbohydrates, in subjects with hypertension (86). Maki et al. (65) reported that diastolic blood pressure during controlled feeding was reduced by a mean of 1.5 mm Hg from baseline with consumption of foods that incorporated 54 g extra-virgin olive oil/d (high in MUFAs), but was unchanged from baseline when subjects consumed the same quantity of foods made with corn oil (high in PUFAs). The Cochrane review by Al-Khudairy et al. (69) reported no significant effects on systolic blood pressure (mean difference −0.79; 95% CI: −3.0, 1.41) or diastolic blood pressure (mean difference −0.02; 95% CI: −1.35, 1.32) in a pooled analysis of 2 clinical trials that increased ω-6 fatty acid intake. The Dietary Intervention and Vascular Function study that examined the replacement of SFAs with either MUFAs or ω-6 PUFAs in men and women with moderate CVD risk also failed to detect effects on flow-mediated dilation and most blood pressure variables, but MUFAs did attenuate the increase in night systolic blood pressure (−4.9 mm Hg, *P* = 0.019) (87). The overall conclusion from these studies is that there might be a small blood pressure–lowering effect of replacing carbohydrates with MUFAs, but there is likely a neutral effect of ω-6 PUFAs on blood pressure.

### Insulin sensitivity

Several clinical trials have reported that exchanging the major classes of dietary fatty acids, i.e., replacing SFAs with PUFAs or MUFAs, favorably affects glucose and insulin metabolism and reduces the risk of T2D (64, 88–90). A systematic review and meta-analysis of 102 controlled trials was recently conducted by Imamura et al. (90) to examine the effects of dietary fatty acids on glucose and insulin metabolism. They reported that substituting 5% of energy from SFAs with PUFAs decreased fasting glucose by a mean of 0.04 mmol/L (95% CI: −0.07, −0.01 mmol/L; *P* < 0.05; 99 trials), lowered HbA1c by 0.15% (95% CI: −0.23%, −0.06%; *P* < 0.001; 23 trials), and lowered the HOMA-IR by 4.1% (95% CI: −6.4%, −1.6%; *P* < 0.05; 30 trials). Based on an HbA1c improvement of ∼0.1% for each 5% increase in energy from PUFAs, the authors predicted that this would translate into a 22% reduction in T2D risk (91).

Experimental evidence supports the biological plausibility of beneficial effects of PUFAs, particularly PUFAs that are predominantly ω-6 LA, on several mechanisms associated with insulin sensitivity and the development of T2D, including, but not limited to, suppressing hepatic lipogenesis, steatosis, and pancreatic lipotoxicity, as well as dampening the toxicity of tissue FFAs, increasing membrane fluidity, positively affecting markers of mitochondrial content and function, and others (90, 92–100). More studies are needed to evaluate the effects of individual fatty acids on insulin sensitivity, which is a major determinant of T2D risk.

### Inflammation

Practically all chronic diseases, including CVD, diabetes, and obesity, are known to manifest some aspects of inflammation; there are also many diseases where chronic inflammation is identified as the central issue, such as asthma, arthritis, and inflammatory bowel disease (101). Recently, the results of the Canakinumab Anti-inflammatory Thrombosis Outcomes Study (CANTOS) provided proof of the inflammatory hypothesis of atherothrombosis, i.e., proof of the concept that reducing inflammation lowers CVD risk (102). CANTOS demonstrated that administration of canakinumab, a monoclonal antibody targeting IL-1β, significantly lowered the rate of recurrent cardiovascular events, compared with placebo, in 10,061 patients with previous myocardial infarction and a high-sensitivity C-reactive protein (hs-CRP) concentration ≥2 mg/L, independent of lipid lowering.

In nearly every discussion about the recommendation to increase dietary PUFA as a replacement of SFA, there is concern expressed about the potential risk of dietary ω-6 PUFAs increasing inflammation (103). ω-6 And ω-3 fatty acids act as competing substrates for some of the same metabolizing enzymes, and a generalization is usually made that anti-inflammatory compounds are produced by ω-3 fatty acids and proinflammatory compounds are produced by ω-6 fatty acids (104–106). The putative risk from ω-6 fatty acids relates primarily to the conversion of LA to AA, a precursor to inflammatory eicosanoids such as prostaglandin E3 and leukotriene B5 (27, 103, 107). However, the lipoxins, specialized proresolving mediators derived from ω-6 fatty acids, act in reducing/resolving inflammatory responses (108). The effects of downstream ω-3 and ω-6 fatty acid products are incompletely understood, and research suggests it is likely that there is more interplay between the pro- and anti-inflammatory processes of these fatty acid subclasses than previously appreciated (109). It has also been suggested that rather than a direct relation between the amount of dietary LA and increased inflammation, the risk may actually be related to an elevated dietary ω-6 fatty acid to ω-3 fatty acid ratio, which in most modern Western diets exceeds 10:1 (27, 110–112).

Despite theoretical and experimental evidence suggesting that increasing ω-6 fatty acid intake increases inflammation, observational studies and RCTs in humans do not support this relation (109). In their examination of data from the Nurses’ Health Study and the Health Professionals Follow-up Study, Pischon et al. (113) reported that the lowest amounts of inflammation were observed in the subjects with the highest intakes of both ω-3 and ω-6 fatty acids. A cross-sectional analysis of Italian adults showed that plasma total ω-6 fatty acid concentrations were inversely associated with several markers of inflammation, including serum CRP, IL-6, IL-6 receptor, IL-1 receptor agonist, and TNF-α (114). A recent cross-sectional substudy from the BALANCE Program Trial of 364 patients with established CVD also demonstrated that PUFAs were inversely associated with CRP concentrations and IL-1β. Increasing 1 g/1000 kcal in PUFAs, ω-3 PUFAs, and ω-6 PUFAs was shown to be associated with reduced mean concentrations of IL-1β of 6%, 48%, and 8%, respectively (115). More recently, a cross-sectional analysis of 1287 healthy men aged 42–60 y from the Kuopio Ischaemic Heart Disease Risk Factor Study, 1984–1989 demonstrated that both serum total ω-6 PUFA and LA concentrations were associated with lower hs-CRP concentrations in multivariable-adjusted analyses (116). Across increasing tertiles of serum LA, mean hs-CRP concentrations were 1.86, 1.51, 1.53, and 1.37 mg/L (*P*-trend = 0.001), and the OR (95% CI) for elevated hs-CRP in the highest compared with the lowest LA quartile was 0.47 (0.25, 0.87, *P*-trend = 0.01). AA, gamma-linolenic acid, and dihomo-gamma-linolenic acid were not associated with higher hs-CRP concentrations.

A systematic review by Johnson and Fritsche (109) of 15 RCTs that assessed the effects of dietary LA on several biological markers of chronic inflammation (C-reactive protein, fibrinogen, plasminogen activator inhibitor-1, cytokines, soluble vascular adhesion molecules, or TNF-α) reported only 2 studies with significant findings: greater excretion of prostaglandin E2 and lower excretion of 2,3-dinor-thromboxane B2 in 1 study, and higher excretion of tetranorprostandedioic acid in another study. Therefore, Johnson and Fritsche (109) concluded that there were virtually no data from RCTs to support the claim that increased consumption of ω-6 fatty acids promotes inflammation in healthy adult humans. Additional long-term RCTs are needed to further evaluate the relation between increased dietary ω-6 fatty acid intake and inflammation. However, the evidence to date does not support an adverse effect on markers of inflammation. Effects of dietary fatty acids on body fat and composition is another related topic of interest, but beyond the scope of this review. The interested reader can learn more about this topic in recently published reviews (117–119).

## Conclusions

To summarize, the data from RCTs regarding the replacement of SFAs with ω-6 PUFAs on CVD outcomes suggest potential benefits, but are, in our view, inconclusive at present. Well-designed and properly controlled RCTs are needed to more clearly elucidate the potential cardiometabolic benefits and possible risks of replacing SFAs (and refined carbohydrates) with ω-6 PUFAs. The epidemiologic evidence base supporting an inverse relation between ω-6 fatty acid intake and CVD, CAD, diabetes, and CVD mortality is larger and generally supports reduced cardiometabolic disease risk with higher intakes of ω-6 PUFAs.

Clinical trial evidence of cardiometabolic markers of risk of CVD and T2D also indicates favorable effects of ω-6 PUFAs on blood lipids and insulin sensitivity, and a neutral effect on blood pressure. Although potential for increased inflammation with increased ω-6 PUFA intake is often raised as a concern regarding dietary recommendations to increase PUFA consumption, observational studies and limited data in humans from RCTs of markers of inflammation do not support an association between inflammation and ω-6 PUFA intake. In conclusion, the results summarized in this review generally support the DGA 2015 recommendation to limit the intake of SFAs to <10% of energy/d by replacing with UFAs (2), and the AHA Presidential Advisory recommendation to shift from SFAs to UFAs, especially PUFAs, in conjunction with an overall healthy dietary pattern (13). However, additional RCTs are needed to more fully evaluate the effects of using ω-6 PUFAs as a replacement for other dietary components such as SFAs, refined starches, and added sugars with evaluation of incident cardiometabolic disease events.

## Notes

Supported by ACH Food Companies, Inc.

Author disclosures: FE and MEC are employees of ACH Food Companies, Inc. KCM and MRD have received research funding and funding to write this manuscript from ACH Food Companies, Inc. MHD is a founder of Omthera Pharmaceuticals, now owned by AstraZeneca.

Abbreviations used:

AA: arachidonic acid
ALA: alpha-linolenic acid
CAD: coronary artery disease
CVD: cardiovascular disease
DGA: Dietary Guidelines for Americans
EPIC: European Prospective Investigation into Cancer and Nutrition
HbA1c: glycated hemoglobin
hs-CRP: high-sensitivity C-reactive protein
LA: linoleic acid
RCT: randomized controlled trial
T2D: type 2 diabetes mellitus
TC: total cholesterol
UFA: unsaturated fatty acid
